# Non-invasive Assay for Measurement of Fecal Triiodothyronine (T3) Metabolite Levels in European Mouflon (*Ovis aries musimon*)

**DOI:** 10.3389/fvets.2022.851794

**Published:** 2022-05-30

**Authors:** Valeria Pasciu, Maria Nieddu, Elena Baralla, Marco Muzzeddu, Cristian Porcu, Francesca Daniela Sotgiu, Fiammetta Berlinguer

**Affiliations:** ^1^Department of Veterinary Medicine, University of Sassari, Sassari, Italy; ^2^Department of Chemistry and Pharmacy, University of Sassari, Sassari, Italy; ^3^Agenzia Regionale FoReSTAS, Centro di Allevamento e Recupero della fauna Selvatica di Bonassai, Sassari, Italy

**Keywords:** triiodothyronine T3 metabolites, feces, mouflon, ELISA, validation

## Abstract

Thyroid hormones (THs) are important indicators of metabolism and animal health. Traditionally, they have been determined from blood or urine samples. However, as their collection may be stressful and requires ethical approval, alternative non-invasive matrices are preferred when dealing with wild animals. Triiodothyronine (T3) is the active form of THs in blood and the major metabolite excreted in feces. This creates the ideal conditions for its assay in fecal samples. Fecal sampling eliminates the stress of the animals and the need to physically capture them. However, in wild species it is rare to find species-specific kits for the hormone assay. So, the objective of this work was to validate a method for the quantification of T3 metabolite (FTM) levels in feces of European mouflon by using an economic and easily available ELISA kit designed to quantify T3 in human plasma. Analytical and biological validations were performed in feces collected from 10 mouflons (5 ewes and 5 rams). An efficient liquid-extraction method was optimized. Precision, dilution linearity, parallelism, recovery and stability of T3 in fecal samples were calculated. Obtained data were considered acceptable according to international guidelines. The reliability of the results was verified comparing human plasma and mouflon fecal samples fortified with the same T3 standard solutions. The biological validation showed higher FTM levels in March compared to June, and no differences between mouflon ewes and rams. The validation of the present method provides a non-invasive and affordable tool for the quantification of FTM in European mouflon.

## Introduction

Thyroid hormones (THs) are the end product of the activation of the hypothalamic–pituitary–thyroidal (HPT) axis. Thyrotropin-releasing hormone (TRH), produced and released by the hypothalamus, stimulate the anterior pituitary gland to secrete thyroid-stimulating hormone (TSH, or thyrotropin) into the bloodstream. In the thyroid gland, TSH stimulates the production of tetraiodothyronine (T4) and triiodothyronine (T3) from thyroid follicles. Only 20% of circulating T3 is directly secreted by the thyroid gland, while 80% is produced by peripheral mono-deiodination from T4 (1, 2). T3 is commonly considered more biologically active and potent than T4, and therefore has greater biological and clinical importance (3).

THs act on many different target tissues, stimulating oxygen utilization and heat production in every cell of the body and playing a role in several physiological processes like metabolic rate, the maintenance of bone health, muscle control, brain function and development, heart and digestive functions. Appropriate thyroid gland function is considered crucial to sustain animal well-being (4) and variation of THs circulating levels allows the animals to adapt their metabolic balance to different environmental conditions, variations in nutrient requirements and availability, and to homeorhetic changes during different physiological stages. The overall effects of THs lead to an increase of the basal metabolic rate, enhancing glucose cells availability, stimulating protein synthesis and lipid metabolism, and finally improving cardiac and neural functions (5). T4 and T3 blood concentrations change with age, sex and seasons (6, 7). Nutritional deficit, the lowering of metabolism and consequent conservation of energy during the nutritional emergency, are effects of T4 and T3 concentrations change (6, 8). Moreover, T4 and T3 measures may also provide a valuable index of climate change impacts among free-ranging wildlife. In fact, their concentration is inversely proportional to the variation of the temperature below or above the thermal zone of the species (9, 10). The attention of the physiology community for measuring THs is consequently high, given the large amount of information that can be obtained on the animal's health state and on its living environment (6, 11).

In recent years, the use of non-invasive techniques for the analysis of endocrinological parameters in animals and humans has been intensified (12, 13). This aspect is essential especially for wild animals since it eliminates the stressful events associated with capture, handling and the need to acquire ethical approval. Traditionally, THs have been determined from blood or urine samples. However, the urine and blood collection requires the capture and restraint of wild animals without considering the stress that would result for them (6, 13). In birds and mammals, THs are excreted in the bile and this create the conditions for their determination in feces where T3 represents the major metabolite, as confirmed by Wasser et al. in dogs (6). The use of feces as matrix allows a safe and easy sampling technique (14). Recent studies have validated and applied protocols using Radio Immuno Assays (RIA) for determination of fecal T3 metabolite (FTM) levels in several species as killer whales (*Orcinus orca*), monk seals (*Monachus schauinslandi*), caribou (*Rangifer tarandus*), northern spotted owls (*Strix occidentalis caurina*), wild impala (*Aepyceros melampus*), wild baboons (Papio ssp) (15–20). It is important to highlight that T3 is a very conserved molecule during evolution (21). RIA is a very sensitive method, but at the same time, it is expensive and needs qualified personnel and equipped laboratories. In addition, radioactive assays are being gradually replaced with assays containing enzymatic labeling in order to decrease the use of radioactive products.

Until now, there are no available specific ELISA kits for FTM determination in wild ungulates and the commercial kits designed for domestic species are very expensive and not always available. In previous studies (12, 22), FTM levels were measured in wild deer fecal samples using a specific kit for T3 in bovine plasma, justifying its use because of the phylogenetically relationship between the wild deer and bovids. However, these kits are very expensive and not as easy to find compared to the more common kits for humans.

Finding alternative assays is an open area of research for wildlife endocrinologist. However, the use of human plasma/serum kits is not common for this aim when analyzing fecal/urine samples. Human kits are indeed often based on monoclonal antibodies and thus have the disadvantage of not being able to detect compound-metabolites.

Starting from these premises, the objective of this work was to create a new, easily available and affordable research tool for studying FTM fluctuations in fecal samples from wild ungulates by verifying whether a human-designed ELISA kit was suitable for FTM quantification in a different matrix and species. In particular, the present study validated the use of an immuno-enzymatic competitive ELISA kit designed for T3 quantification in human blood samples for the quantification of FTM in European mouflons (*Ovis aries musimon*). The kit used in this study is based on group-specific polyclonal antibodies and thus it allows the identification not only of the mother compound, but also of compound-metabolites, often present in non-invasive matrices.

## Materials and Methods

### Materials

Triiodothyronine (T3) standard and ethanol was obtained from Merck (cod. 642511 and 51976); T3 ELISA Kit was obtained from Diametra (DiaMetra Srl Management and Coordination: Immunodiagnostic Systems (IDS) Ltd, Boldon, UK, a PerkinElmer Company). This kit is based on group-specific polyclonal antibodies able to identify the mother compound and its metabolites. The lower detection limit of the kit (0.05 ng/mL) was suitable for our analytical purpose. The specific cross-reactivity supplied by the manufacturer for the T3 antibody to other compounds was: l-triiodothyronine 1.00%, d-triiodothyronine 0.015%, l-thyroxine 0.01%, d-thyroxine 0.0025%, monoido-tyrosine n.d., diiodo-tyrosine n.d., triiodothyroacetic acid n.d. and tetraiodothyroacetic acid n.d.

European mouflons were permanently housed in an outdoor pen at the Wildlife Rescue Center within a reproduction in captivity program (Centro Allevamento e Recupero Fauna Selvatica di Bonassai–Forestas). Animals were fed concentrate feed mixture and roughages with freely available water and housed in semi-open sheds, allowing free grazing. Fecal samples were recovered after posting and observation of the animal's defecation, without causing animal stress. Fecal samples were collected from adult (3–5 years old) individuals (ewes and rams) in a good body condition and health status. The mouflon ewes tested in this study were not pregnant.

No permission or authorization from the ethics committee was required for fecal samples collection. The fecal samples were collected with surgical sterile gloves and were put into sterile centrifuge tubes, sealed and marked and transported on ice to laboratory within 2 h from collection. Pooled human plasma was purchased from Innovative Research (Novi, MI). No institutional regulations about the use of blood from these sources were required. No human subjects were used in this research.

### T3 Extraction From Feces

For this study, fecal samples from 10 European mouflons (5 ewes and 5 rams) were collected. Fresh samples were homogenized individually and freeze-dried in 15 mL tubes as described in literature with some modifications (6, 22). The lyophilized sample was then treated with different volumes of ethanol to optimize the extraction conditions. The increase in cycles and time extraction and the decrease in the percentage of ethanol from 98% to 70% allowed a better recovery, as already reported by Wasser et al. (6).

In detail, 0.2 g of unfortified feces (UF) were used to determined basal FTM concentrations. Then, 0.2 g of feces fortified with 10 ng of standard T3 (FF10) and 0.2 g with 50 ng of standard T3 (FF50) were used to obtained spiked samples. All samples were freeze-dried and extracted, as reported below: 2 ml of 70% ethanol was added to the lyophilized feces, kept for 30 min under agitation and then, the samples extracted were centrifuged at 2500 rpm for 20 min. The supernatant was recovered in a tube and the fecal pellet was re-extracted (2 mL of 70% ethanol) two more times. The three extracted fractions were combined and dried under stream of nitrogen; dry residue was reconstituted with 1 ml of phosphate buffered saline (PBS).

### ELISA Assay

The reconstituted samples were analyzed with T3 ELISA kit using a microplate reader (POLARstar Omega; BMG Labtech), with BMG Labtech software for data analysis. The assay is a competitive method, where the antigen (T3 in the sample) competes with the antigenic T3 conjugated with horseradish peroxidase (HRP) for binding to the limited number of antibodies anti-T3 coated on the microplate (solid phase). Quality controls provided by the manufacturer were used to verify the performance of the assay. After incubation, the bound/free separation is performed by a simple solid-phase washing. Then, the enzyme HRP in the bound-fraction reacts with the TMB Substrate and develops a blue color that changes into yellow when the Stop Solution (H_2_SO_4_) is added. The color intensity is inversely proportional to the T3 concentration of the sample. T3 concentration is calculated through a calibration curve (0-7.5 ng/mL).

### Analytical Validation

For analytical validation precision, dilution linearity, parallelism, recovery, and samples stability were measured. The precision of the method, evaluated at the two concentrations (10 and 50 ng/mL), was expressed as the percent relative standard deviation (RSD %) and calculated for five replicates for the intra-day repeatability and over three consecutive days for the inter-day repeatability. Dilution linearity (for fortified samples) and parallelism (for unfortified samples) allow to verify whether the assay maintains linearity under dilution (23) and were expressed as coefficient of variation (% CV). The accuracy, which represents the closeness of the test results to the true values, was determined as recovery % for five replicates using the following formula:


$$\frac{\text{F}\text{F}}{\text{S}\text{T}\text{D}}\times 100\;$$

Where, FF was FF10 or FF50, and STD was T3 standard solution at 10 or 50 ng/mL in PBS.

The freeze-and-thaw stability of T3 in the fecal samples was determined after performing three freeze-and-thaw cycles at two tested concentrations (10 and 50 ng/mL). The samples (n = 5) were first analyzed as fresh, and then frozen for 24 h and thawed for 1 h at room temperature. This freeze-and-thaw cycle was performed three times. The long-term stability was determined on the same samples after 30 days of storage at −20°C.

### Comparison of the Response of T3 in Human Plasma *vs*. Mouflon Fecal Samples

The reliability of the results in fecal samples was verified by fortifying both human plasma and mouflon feces with the same T3 standards. Extracts of unfortified mouflon feces (UF) were fortified with T3 standard solution to obtain a final concentration of 10 and 50 ng/mL, respectively (MF10, *n* = 5 and MF50, *n* = 5). The basal T3 concentration in mouflon fecal samples, measured in UF, was subtracted from MF10 and MF50. These values were compared with the corresponding T3 standard solutions at 10 and 50 ng/mL. Unfortified human plasma (HP) and human plasma fortified with 10 and 50 ng/mL (HP10, *n* = 5 and HP50, *n* = 5) were analyzed as reported by the kit manufacturer. The basal T3 concentration in human plasma (HP) was subtracted from HP10 and HP50 and the obtained values were compared with the corresponding T3 standard solutions at 10 and 50 ng/mL. Finally, results in human plasma and mouflon feces were compared to evaluate the response of the kit in the two different matrices.

### Biological Validation

To perform the biological validation of the method, we compared differences in FTM levels between mouflon ewes (*n* = 5) and rams (*n* = 5) and in samples collected from the same animals in two different periods: July (Average Ambient Temperature= 26°C, Tmin = 17°C, Tmax = 33°C) and March (average ambient temperature= 11°C, Tmin = 3°C, Tmax = 19°C). One sample per animal was collected at each sampling period.

### Statistical Analysis

Results are expressed as mean values (mean ± SD). Variable normality of the studied groups (analytical validation: T3 standard, human plasma and mouflon feces; biological validation: female and male mouflon feces collected in July 2021 and March 2022) were assessed by the Kolmogorov–Smirnov test. Differences were considered to be statistically significant at *p* < 0.05.

Differences between groups of the analytical validation, either for the 10 and 50 ng/mL concentration, were analyzed by a One-way ANOVA, using the groups as factor, in order to point out statistical differences between the standard and the two biological matrices. As *post-hoc* test, Tukey's test was used to highlight possible differences within and between groups.

For the biological validation, T3 levels in fecal samples were compared using paired and unpaired *T*-tests. Paired *T*-test were performed to highlight differences within groups (female and male) during time (July and March); unpaired *T*-test were used to highlight differences between groups in each month. Alpha levels were adjusted using Bonferroni correction.

Analyses were performed using Minitab 17 Statistical Software (2010, Minitab, Inc., State College, PA, USA).

## Results and Discussion

Data validation on fecal samples showed good accuracy with recovery values of 81.4% and 72.8% for 10 and 50 ng/mL, respectively (Table 1). Recovery rates was acceptable according to international guidelines (70–120%) (24). The repeatability data were within 15% as requested by the guidelines for method validation (Table 1) (25). The dilution linearity and parallelism were within acceptable values (% CV <30%) (23). Parallelism determination allowed to confirm that the binding of the endogenous analyte to the antibodies was the same as for the calibrator. Regarding stability results, the loss observed was lower than 10%, as shown in Table 1.

**Table 1:** Repeatability, recovery, and stability of triiodothyroxine (T3) measurement in fecal samples of European mouflons.

	**T3**	**Repeatability (RSD%)**		**Recovery**	**Stability (%** **±** **SD)**	
**Sample**	**concentration**	**intra-day inter-day**		**(% ± SD)**	**Freeze-thaw**	**30-days**
Feces	10 ng/mL	6.88	8.98	81.4 ± 10.3	98.3 ± 8.9	91.5 ± 5.7
	50 ng/mL	7.68	6.96	72.8 ± 6.2	99.0 ± 4.7	97.4 ± 2.3

*RSD%, relative standard deviation*.

Taken together, our results showed that FTM levels can be accurately measured in European mouflon and are stable under storage conditions used for hormone analysis. Data stability is very important in field studies. It is often necessary to store samples at−20°C before carrying out the analysis, therefore, it was essential to verify samples stability for at least 1 month at −20°C.

Furthermore, as additional verification on the applicability of the ELISA kit on fecal samples, human plasma and mouflon feces were fortified with the same standard solutions of T3. The response of the two fortified matrices was comparable when considering both 10 and 50 ng/mL standards (Table 2).

**Table 2:** Comparison of T3 concentration between T3 standard, human plasma and mouflons feces, both fortified with T3 standard (10 and 50 ng/mL).

**Nominal concentration**	**T3 standard**	**Human plasma**	**Mouflons feces**
**(ng/mL)**	**concentration (ng/mL) (mean ± SD)**	**(ng/mL)**°** (mean ± SD)**	(ng/mL)* (mean ± SD)	***p*-Values**
10	10.10 ± 0.30	10.21 ± 0.95	10.44 ± 1.36	0.135
50	49.96 ± 1.62	50.56 ± 1.69	51.19 ± 2.97	0.213

Basal values were subtracted from fortified samples. °No fecal samples were available; *No plasma samples were available.

The biological validation did not show differences in FTM levels between mouflon ewes and rams, thus confirming previous findings in wild ungulates (26, 27). As expected, FTM levels varied according to the ambient temperature, being higher in both sexes in March compared to July (Table 3; *p* < 0.0001). This preliminary result confirms that ambient temperatures is a main driver in T3 fluctuations in wild ungulates (27). Moreover, the obtained FTM levels were comparable with those reported in literature for another wild ungulate species (12, 22).

**Table 3:** Differences in fecal T3 metabolites (FTM) levels between mouflon ewes (*n* = 5) and rams (*n* = 5) in two different periods: July and March.

**Sex**	**FTM levels**	**July**	**March**	**p-Values**		
				**Within the same sex**	**Between sexes**	
					**July**	**March**
Female	ng/mL (ng/g)	3.99 ± 0.59 (19.95 ± 2.95)	8.23 ± 1.17 (41.15 ± 5.85)	*p* < 0.0001	*p* = 0.099	*p* = 0.220
Male	ng/mL (ng/g)	3.41 ± 0.33 (17.05 ± 1.65)	9.29 ± 1.32 (46.45 ± 6.6)	p <0.0001		

*Data are expressed in both measurement units: ng/mL and ng/g of wet feces*.

In this study, the tested ELISA kit, even if designed for human plasma, was found to be suitable for the determination of FTM in European mouflons. This finding provides a new tool for the study of T3 fluctuation in these species. Monitoring fecal T3 can indeed convey important information on the adaptation of the species to varying environmental conditions in relation to sex, age or nutritional status. Interesting studies on the variations of fecal T3 are published in literature (7, 12, 22) for other wild and non-wild species. For example, the monitoring of changes in fecal T3 relating to time of day, sex, reproductive status (28) age and parasitic infections (22), to intestinal microbiota (7) to seasonally cold-habitat (12) was studied, confirming how the secretion of THs change in response to adverse environmental conditions. Affordable analytical methods are a useful tool also for the evaluation of herd health in zoos, game reserves and wild animals. Furthermore, the use of fecal matrices resolves the rising concern about animal welfare (29).

This study can thus contribute to extend our knowledge on the specie studied, adding information to those already present in the literature (29–31).

## Conclusion

In conclusion the method proposed in the present study can be applied for studying FTM levels in European mouflons. Further studies are needed to extend the use of this method on other species, thus resolving its main limitation of being specific for this matrix and species. The validation of an assay significantly less expensive than RIA and HPLC (6, 12, 32), easily available (because for human use) and applicable to non-invasive matrices is of great interest for the scientific community because it can contribute to extend our knowledge on the physiological responses to changing environmental conditions in wild ungulates.

## Data Availability Statement

The raw data supporting the conclusions of this article will be made available by the authors, without unduereservation.

## Ethics Statement

Ethical review and approval was not required for the study on human participants in accordance with the local legislation and institutional requirements. Written informed consent for participation was not required for this study in accordance with the national legislation and the institutional requirements. Ethical review and approval was not required for the animal study because no authorization from the ethics committee was required for fecal samples collection.

## Author Contributions

VP, MN, EB, and FB designed the study. MM, CP, and FDS collected fecal samples. VP performed the experiments and prepared the manuscript. VP, MN, EB, CP, and FB analyzed the data. CP, FDS, MN, EB, MM, and FB revised the manuscript. All authors contributed to the article and approved the submitted version.

## Funding

This work was funded by the University of Sassari (Fondo di Ateneo per la ricerca 2019) and the “Attrazione Mobilità dei Ricercatori” PON AIM 1887720-1 CUP J54I18000160001.

## Conflict of Interest

The authors declare that the research was conducted in the absence of any commercial or financial relationships that could be construed as a potential conflict of interest.

## Publisher's Note

All claims expressed in this article are solely those of the authors and do not necessarily represent those of their affiliated organizations, or those of the publisher, the editors and the reviewers. Any product that may be evaluated in this article, or claim that may be made by its manufacturer, is not guaranteed or endorsed by the publisher.
